# Supplementary material for: Preselection of QTL markers enhances accuracy of genomic selection in Norway spruce
Source: BMC Genomics. 2023 Mar 27;24:147. doi: 10.1186/s12864-023-09250-3 (PMC10041705; doi:10.1186/s12864-023-09250-3)
Supplement: Supplementary file 1 — Additional file 1. Supplementary figures [file 12864_2023_9250_MOESM1_ESM.docx]

**Legends of supplementary Figures.**

**Fig. S1** LD decay (*r*^2^) in a full-sib progeny population (F1-generation, *n*=904, 49 parents in the original pedigree) based on the Norway spruce 50k SNP array. The Vertical dashed line represents LD decay distance when *r*^2^=0.2. The blue curve was fitted by the Hill and Weir formula (Hill and Weir, 1988). In order to visualize the region of LD decay distance when r^2^=0.2, only the distance range from 0 to 2000 kb is shown.

**Fig. S2** Population structure for the 904 clones coloured based on the family identities visualized by marker-based principal component analysis (PCA).

**Fig. S3** Quantile-Quantile (QQ) plots and genomic inflation factors (IFs) for genome-wide association analyses of the six studied traits. The initial slope of the QQ datapoints (black) presents the genomic inflation factor and coincides very closely with the red line (slope=1 and intercept=0).

**Fig. S4** The mating design of 49 parents crossed to generate 32 full-sib families according to the original pedigree. Each circle in the top level represents a parent and the number is the identity of the parent. Each square at the bottom represents a full-sib family. The number within each square represents the number of clones per family genotyped. The families whose squares are framed in red colour, were selected to test the effect of family size on predictive ability. Parents only used for one cross are depicted in dark goldenrod if used as a male and dark skyblue if used as a female. Otherwise, the colour of the parents could be any of both colours.


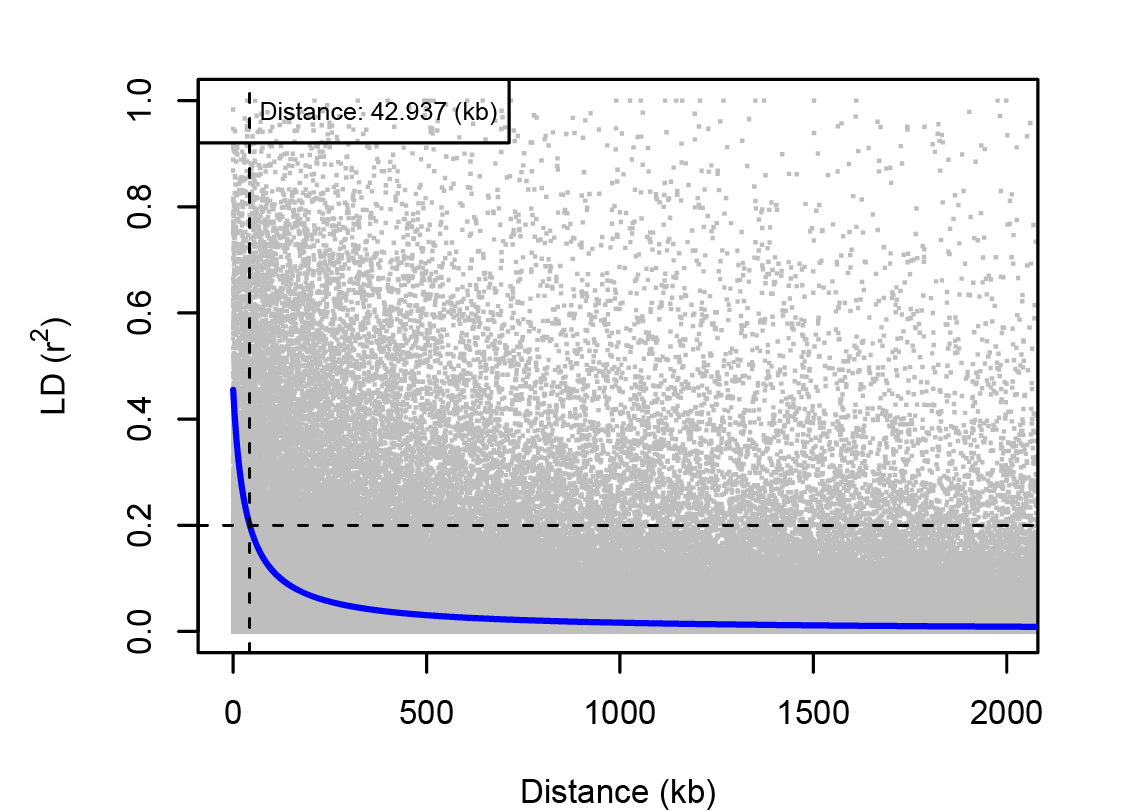


Fig. S1 LD decay (*r*^2^) in a full-sib progeny population (F1-generation, *n*=904, 49 parents in the original pedigree) based on the Norway spruce 50k SNP array. The Vertical dashed line represents LD decay distance when *r*^2^=0.2. The blue curve was fitted by the Hill and Weir formula (Hill and Weir, 1988). In order to visualize the region of LD decay distance when r^2^=0.2, only the distance range from 0 to 2000 kb is shown.


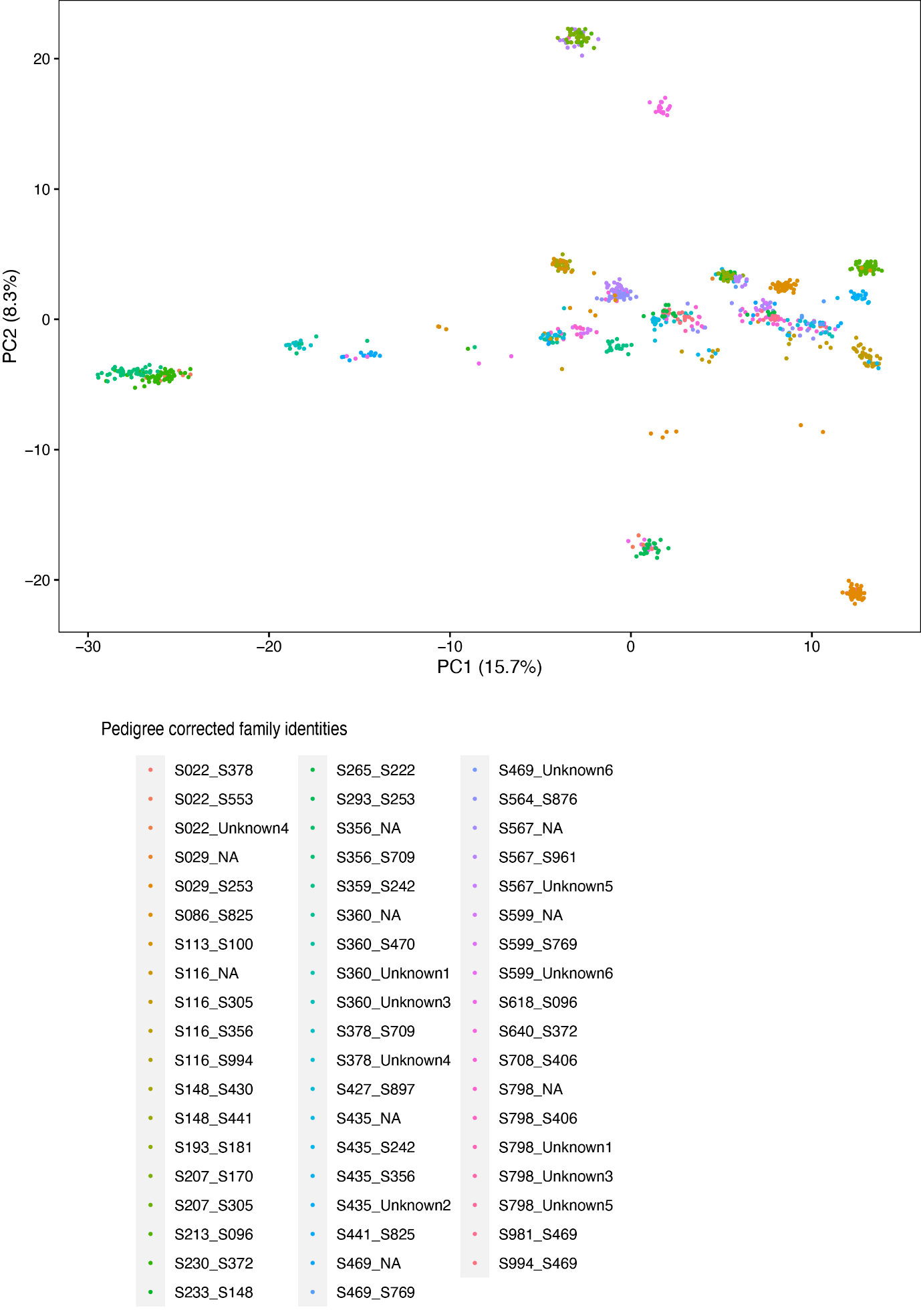


Fig. S2 Population structure for the 904 clones coloured based on the family identities visualized by marker-based principal component analysis (PCA).


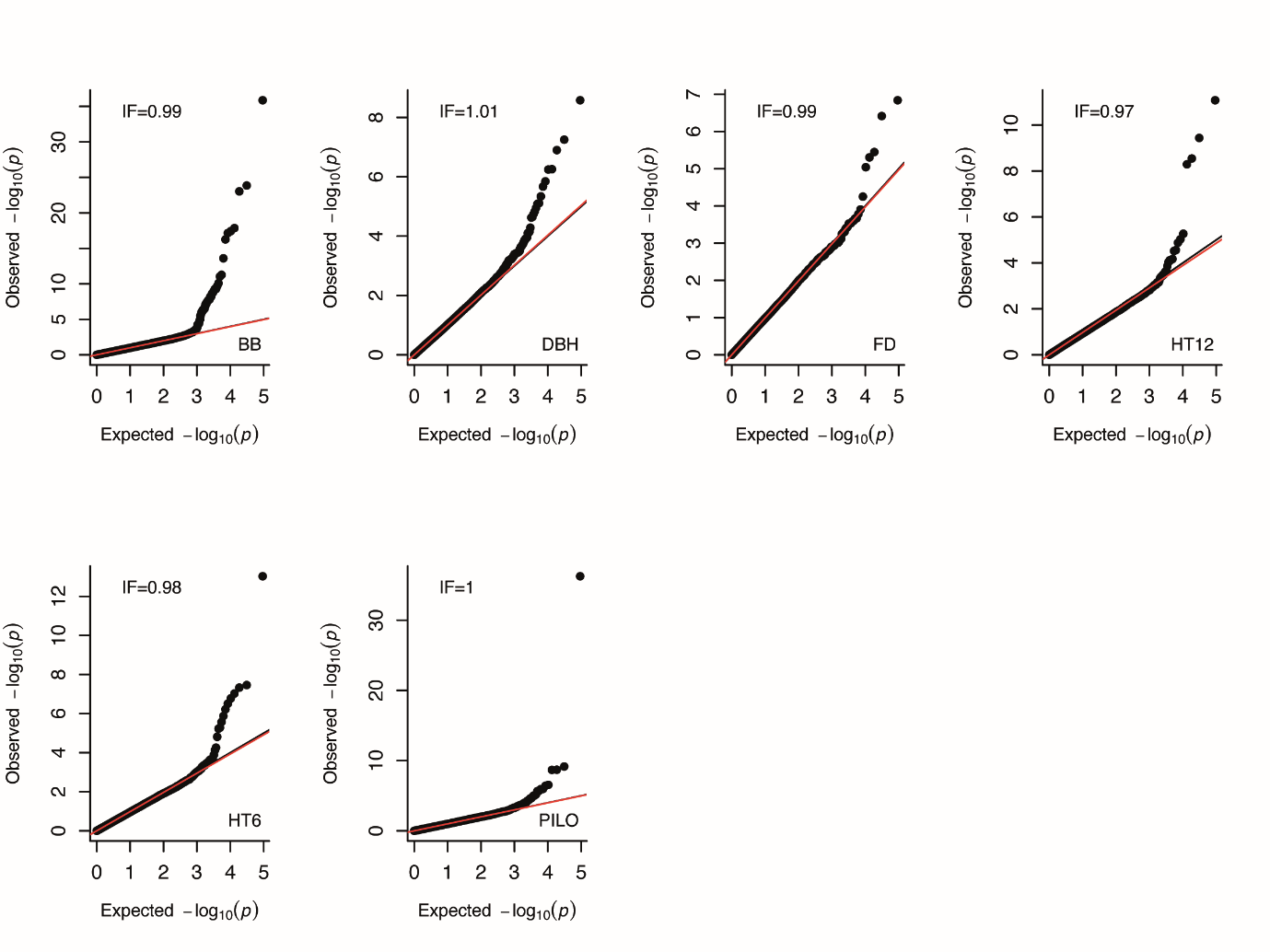


**Fig. S3** Quantile-Quantile (QQ) plots and genomic inflation factors (IFs) for genome-wide association analyses of the six studied traits. The initial slope of the QQ datapoints (black) presents the genomic inflation factor and coincides very closely with the red line (slope=1 and intercept=0).


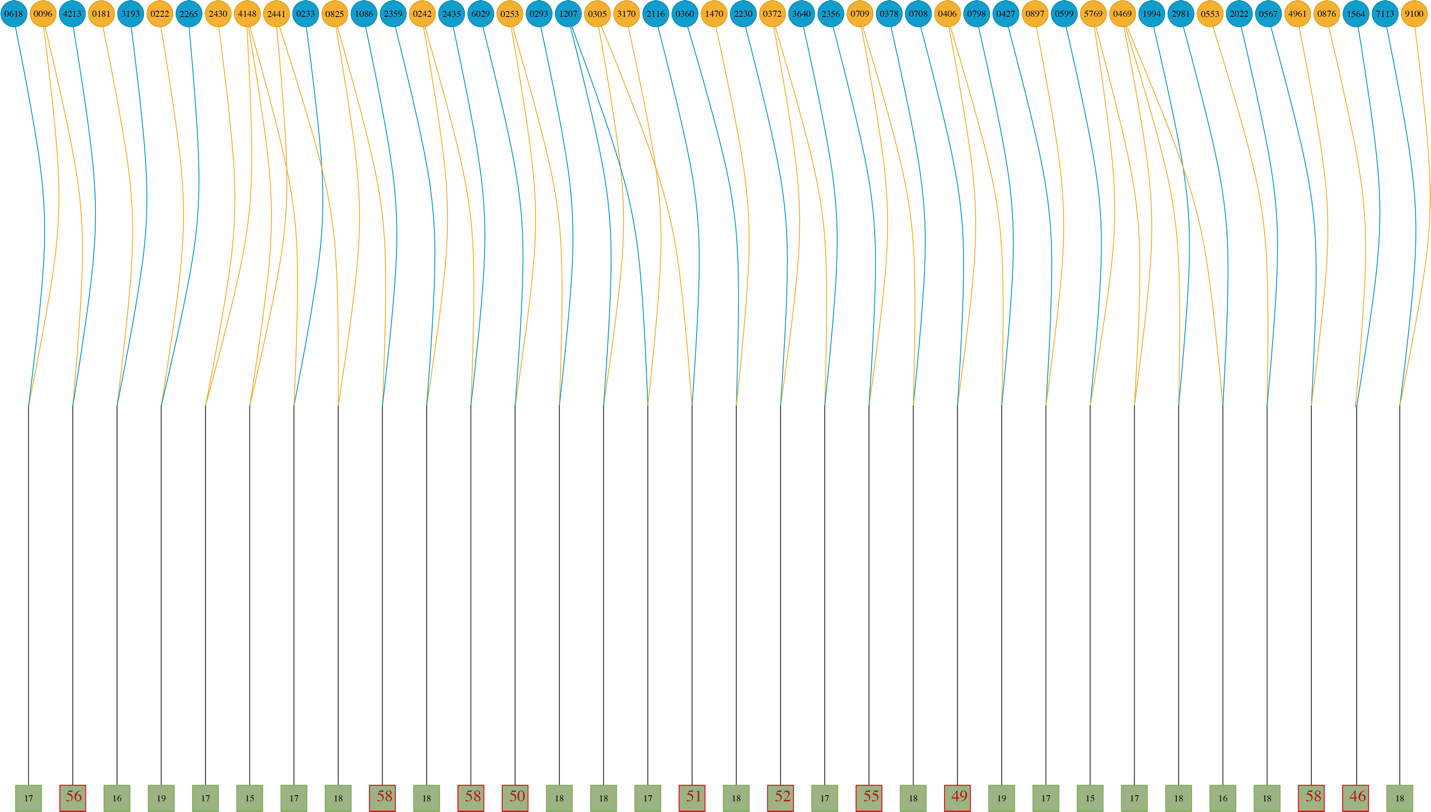


Fig. S4 The mating design of 49 parents crossed to generate 32 full-sib families according to the original pedigree. Each circle in the top level represents a parent and the number is the identity of the parent. Each square at the bottom represents a full-sib family. The number within each square represents the number of clones per family genotyped. The families whose squares are framed in red colour, were selected to test the effect of family size on predictive ability. Parents only used for one cross are depicted in dark goldenrod if used as a male and dark skyblue if used as a female. Otherwise, the colour of the parents could be any of both colours.
